# Neoadjuvant immunotherapy combined with chemotherapy for locally advanced squamous cell lung carcinoma: A case report and literature review

**DOI:** 10.1515/biol-2021-0083

**Published:** 2021-08-25

**Authors:** Yuan Zheng, Wei Zhu, Xinjie Huang, Dongqun Lin, Yu Lin

**Affiliations:** Cardiothoracic Surgery Department, The Second Clinical College of Guangzhou University of Chinese Medicine, Guangdong Provincial Hospital of Chinese Medicine, Guangzhou, Guangdong, 510006, China

**Keywords:** durvalumab, chemotherapy, neoadjuvant immunotherapy, locally advanced resectable NSCLC, case report

## Abstract

The benefit of immunochemotherapy in treating resectable locally advanced non-small cell lung cancer (NSCLC) is not well established. Here, we report a case of resectable stage III NSCLC treated with neoadjuvant immunotherapy combined with chemotherapy before surgery. A 61 years old man was admitted to our hospital due to paroxysmal cough and was diagnosed as squamous cell carcinoma T4N2M0 in the upper lobe of the right lung, which was locally advanced and resectable. He was treated with 3 courses of paclitaxel 250 mg intravenous (IV), carboplatin 0.65 g IV, and durvalumab 620 mg IV followed by thoracoscopic upper lobectomy and lymph node dissection. There was considerable regression of the tumor before surgery, and the patient achieved a complete pathological response after surgery. Our case study demonstrates the benefit of durvalumab and chemotherapy in the treatment of resectable locally advanced NSCLC.

## 1 Introduction

Lung cancer is the leading cause of cancer-related mortality in both men and women worldwide [1,2,3]. The standard treatment options for lung cancer include surgery, radiation therapy, chemotherapy, targeted therapy, and immunotherapy [4]. Surgical resection is the potential treatment option for lung cancer at early stages, whereas multidisciplinary approaches are used during advanced stages [5,6]. Around one-third of the patients diagnosed with lung cancer would have reached stage III, locally advanced disease, when diagnosed with non-small cell lung cancer (NSCLC) [7]. There was heterogeneity in the 5-year survival rate of stage III NSLC with only 36% in stage IIIA, while with 26% in stage IIIB [8]. Postoperative recurrence and metastasis are the main reasons for the low 5-year survival rate in patients with stage III NSCLC [9].

Despite curative resection in patients with NSCLC, 30–70% of patients will experience relapse resulting in death [6,10]. For locally advanced NSCLC, perioperative neoadjuvant therapy and adjuvant therapy are the better treatment choices to improve the prognosis of patients with surgery [2,6,11]. Multimodal approaches such as neoadjuvant chemotherapy, surgery, adjuvant chemotherapy, and radiotherapy are recommended to manage patients with resectable locally advanced NSCLC patients [8]. The survival rate was comparatively higher in NSCLC patients treated with multimodal approaches as compared to patients treated with surgery alone [8,12]. Immunotherapy and targeted therapy are currently new treatment options for NSCLC with positive treatment outcomes [3,8,13,14]. PACIFIC study showed that immunotherapy (durvalumab) combined with radiotherapy can significantly improve the long-term survival rate in patients with unresectable stage III lung cancer, thus becoming a practice-changing study and led to FDA approval [15]. However, there is no enough evidence-based data for the treatment of patients with stage III resectable NSCLC. A phase II multicenter exploratory study reported complete pathological response (CPR) in 71% of the patients, suggesting that immunotherapy combined with chemotherapy might provide positive treatment outcomes as neoadjuvant therapy in patients with resectable stage IIIA NSCLC [16]. However, no studies are evaluating the efficacy of neoadjuvant durvalumab + chemotherapy in resectable stage IIIA NSCLC prior to surgery to our knowledge, and hence, this case is unique. Here, we report and discuss a case of resectable stage III NSCLC treated with neoadjuvant immunotherapy combined with chemotherapy.

## 2 Case presentation

A 61 years old man (body weight: 62 kg; performance score (PS) score: 0–1) was admitted to our hospital on April 10, 2020, due to paroxysmal cough for 6 months. There was no complaint of having expectoration, hemoptysis, chest pain, hot flashes, and night sweats. The patient had a paroxysmal cough for 6 months, and he left it undiagnosed. On April 1, 2020, the patient underwent chest computed tomography (CT) examination as suggested by a local hospital physician. The CT scan reported a space of about 7.9 cm × 6.7 cm × 6.0 cm in the right upper lobe of the lungs. He was admitted to our hospital for treatment. He was a heavy smoker (20 cigarettes per day for 40 years) with no family history of cancer. The patient’s medical report during admission showed that the case was diagnosed as lung cancer [17].

**Figure 1:**
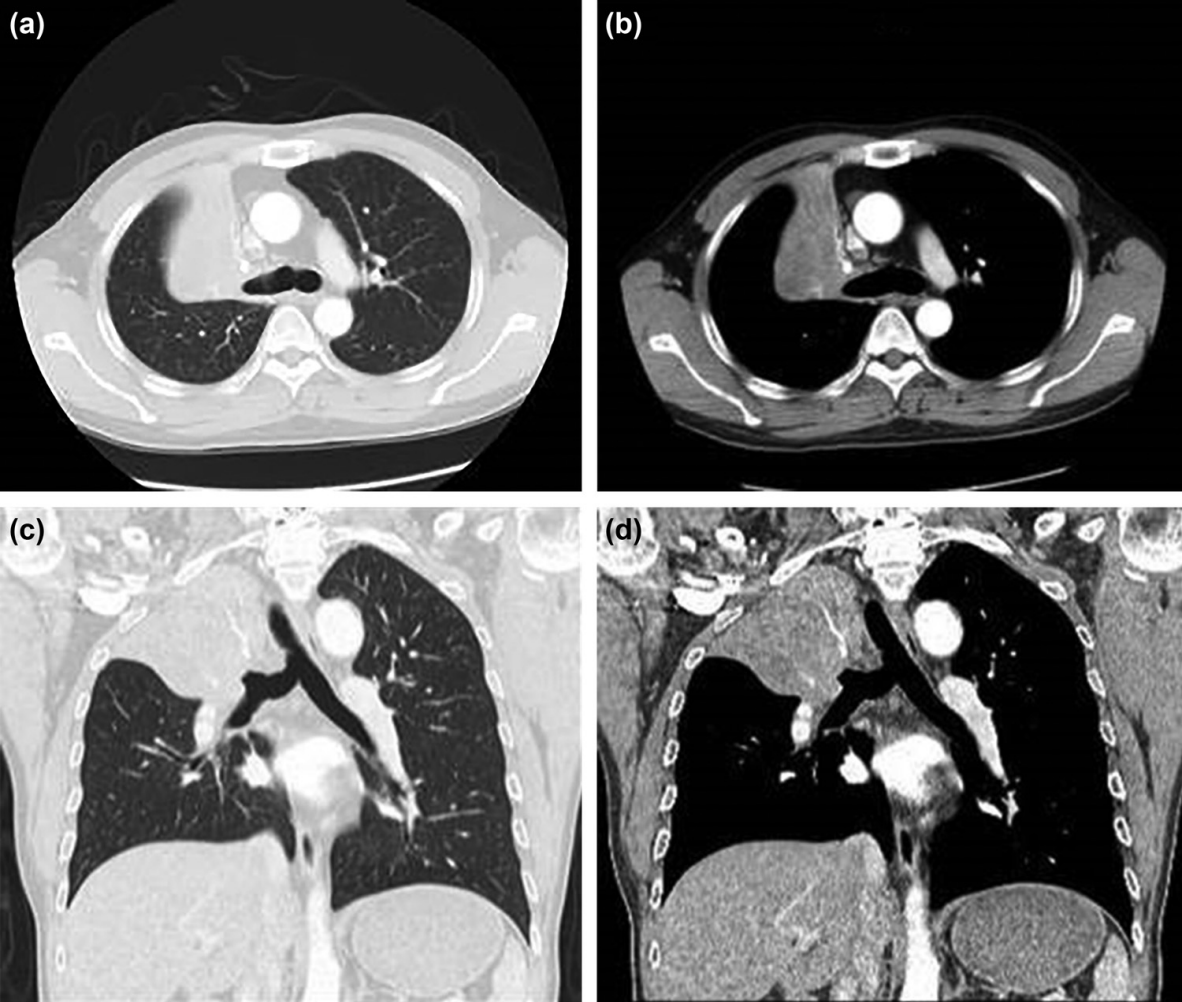
Chest CT scan of the right upper lobe of the bronchus. The right upper lobe bronchus was occluded and the surrounding soft tissue mass was about 7.9 cm × 6.7 cm × 6.0 cm.

**Figure 2:**
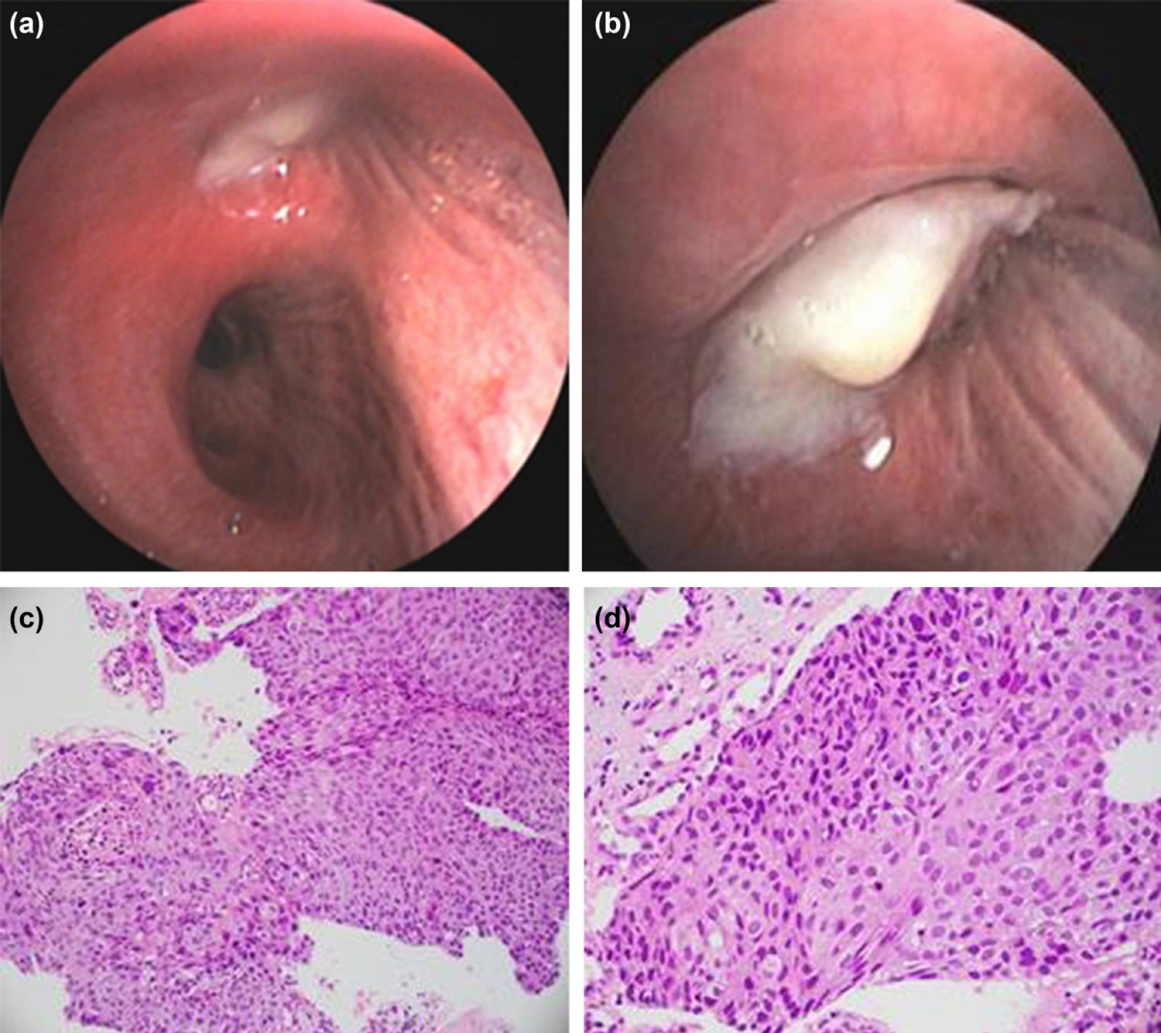
Images of fiberoptic bronchoscopy and histopathology. (a and b) Obstruction of the right upper bronchial lobe. (c and d) Histopathology of fibrobronchoscopy showed squamous cell carcinoma. Immunohistochemistry: TTF-1 (−), p63 (+), CD5/6 (+), P40 (+), and CK7 (−).

On physical examination during admission, there was no swelling of superficial lymph nodes in the whole province. During chest examination, the respiratory sounds of the right upper lung were diminished, and the respiratory sounds of the other parts of the lungs were clear. No dry and wet rales were found in both lungs. The thetumor marker levels in the serum were neuron-specific enolase (NSE) 12.5 ng/mL, squamous cell carcinoma antigen (SCC Ag) 1.7 ng/mL, and cytokeratin 19 fragments 15.8 ng/mL. On April 13, 2020, a chest CT examination showed bronchial obstruction in the right upper lobe surrounded with soft tissue mass, with a size of about 7.9 cm × 6.7 cm × 6.0 cm (Figure 1). Based on the diagnostic assessment, this condition was considered a central type of lung cancer in the right upper lobe, with suspected atelectasis of the right upper lobe. Multiple lymph nodes in the right hilar and mediastinum (2R, 4R areas) were enlarged, and the enhanced scan showed slight enhancement. No metastatic lesions were observed during whole-body bone imaging, brain magnetic resonance imaging, adrenal color Doppler ultrasound, cervical lymph node color Doppler ultrasound, and liver color Doppler ultrasound. Fiberoptic bronchoscopy also revealed a bronchial obstruction in the right upper lobe of the lung (Figure 2a and b). Histopathological examination of the tumor in the upper lobe of the right lung showed a squamous cell carcinoma (Figure 2c and d). Immunohistochemistry assay was TTF-1 (−), p63 (+), CD5/6 (+), P40 (+), and CK7 (−). Hence, the patient was diagnosed with locally advanced lung carcinoma T4N2M0 in the upper lobe of the right lung.

Sleeve resection and lymph node dissection or neoadjuvant chemotherapy plus sleeve resection or radical radiotherapy was suggested to the patient. Based on the patient and his family’s consent, neoadjuvant immunotherapy combined with chemotherapy before surgery was considered. The first course of treatment was paclitaxel 250 mg intravenous (IV) + carboplatin 0.65 g IV and durvalumab 620 mg IV was given along with chemotherapy. On May 13, 2020, during re-examining the tumor markers, NSE was 13.8 ng/mL, SCC Ag was 1.4 ng/mL, and the cytokeratin 19 fragment was 1.9 ng/mL. The second course of treatment was carried out on May 14, 2020.

**Figure 3:**
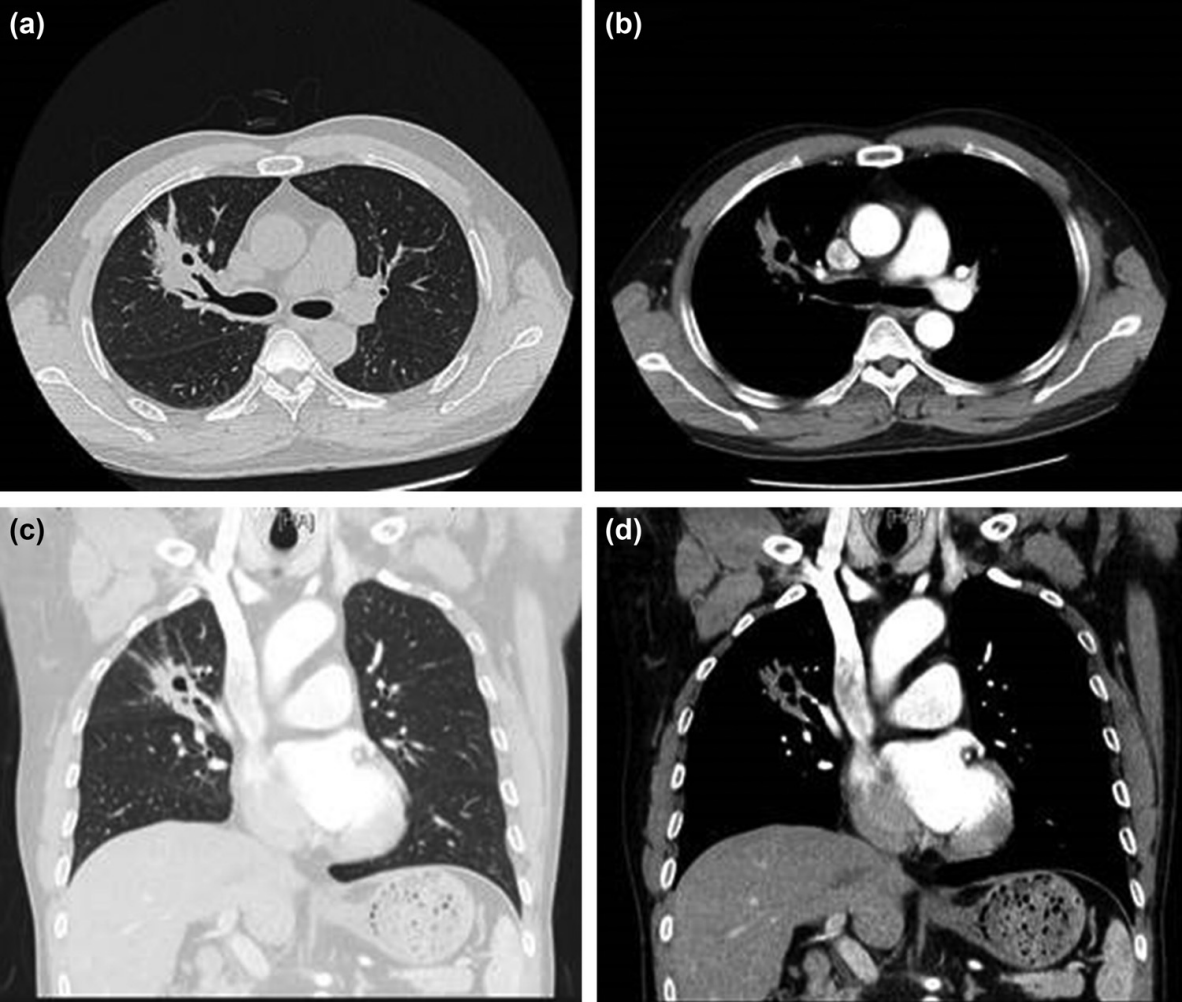
Images of chest CT enhanced scan. (a,b) Bronchial stenosis and occlusion in the right upper lobe of the lung. (c,d) Histopathology of enhanced CT showed soft tissue mass shadow, the size was about 4.2 cm × 2.6 cm × 2.2 cm, the lesion was smaller and the necrotic area was larger compared to previous scan.

On June 10, 2020, the patient was subjected to chest CT enhanced re-examination. The CT scan showed the presence of bronchial stenosis and occlusion in the right upper lobe along with soft tissue mass shadow with a size of about 5.0 cm × 3.2 cm × 2.8 cm (Figure 3). The upper lobe of the right lung expanded than before, multiple lymph nodes in the right hilar and mediastinum (2R, 4R) increased, enhanced scan slightly strengthened, decreased, and shrunk compared with the previous scan. The third course of treatment was started on June 11, 2020. On July 8, 2020, the tumor marker levels were NSE 22.8 ng/mL, SCC Ag 0.8 ng/mL, and cytokeratin 19 fragments 3.1 ng/mL. Chest CT enhanced scan showed right upper lobe bronchial obstruction along with soft tissue mass shadow with a size of about 4.2 cm × 2.6 cm × 2.2 cm. The lesion was smaller, and the necrosis was more extensive than before. Multiple lymph nodes in the right hilar and mediastinum (2R, 4R) were enlarged and slightly enhanced on an enhanced scan, which was nearly the same as before.

**Figure 4:**
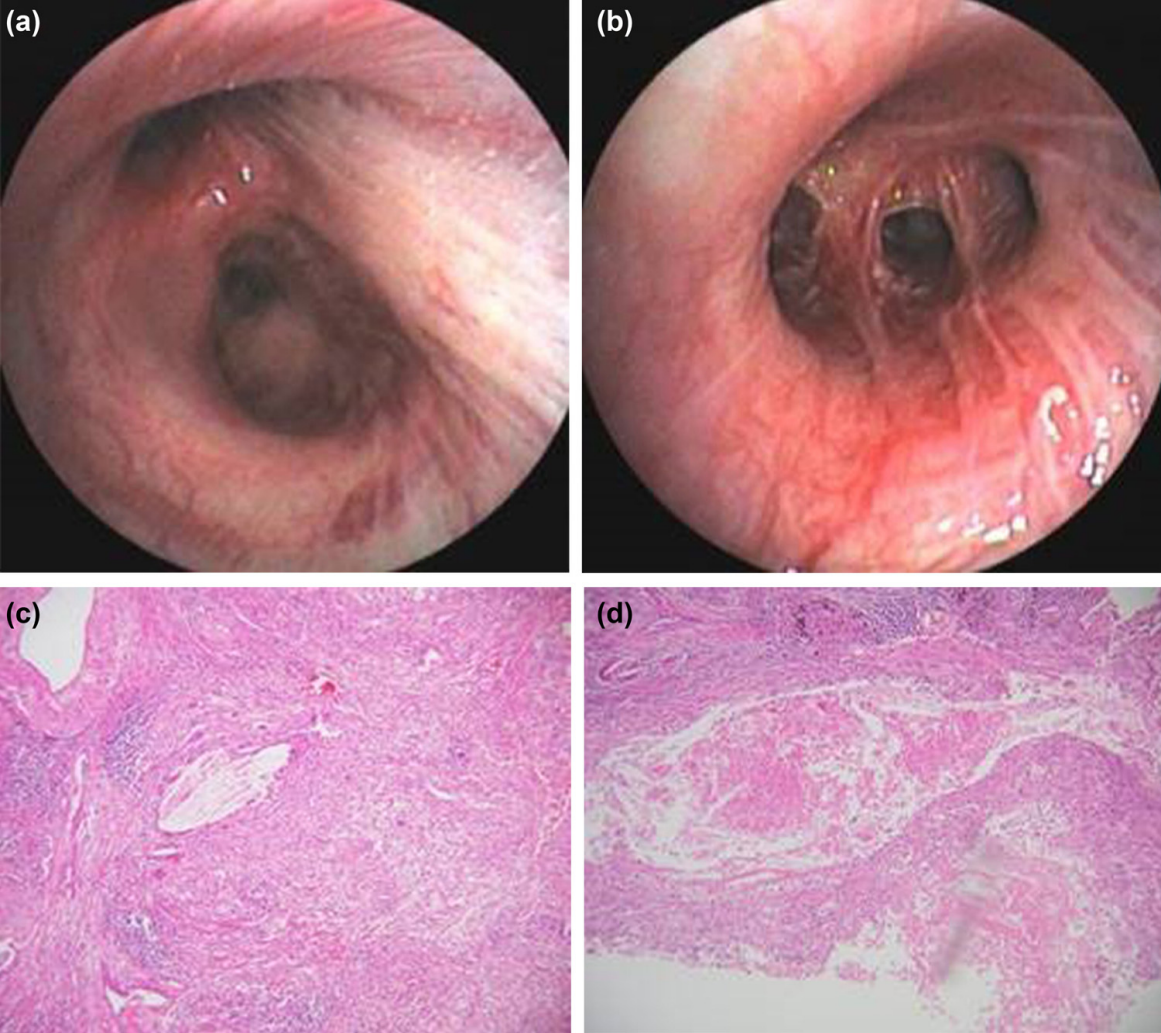
Images of fiberoptic bronchoscopy and histopathology post-treatment. (a and b) Right upper lobe bronchus unobstructed, tracheobronchial bronchitis. (c and d) Histopathology of the tumor after surgery showed necrosis, histiocyte reaction, cholesterol crystal, inflammatory cell reaction under a microscope, and no residual tumor cells.

The clinical efficacy evaluation performed on July 9, 2020, with fiberoptic bronchoscopy showed that after treatment with neoadjuvant immunotherapy and chemotherapy, the right upper lobe of the right lung, the bronchus of the right upper lobe, was unobstructed, and tracheobronchitis occurred (Figure 4a and b). In addition, a biopsy at the opening of segmental bronchus showed chronic inflammation of bronchial mucosa with no cancer.

After considerable tumor regression, thoracoscopic upper lobectomy and lymph node dissections were performed on July 14, 2020. During the surgery, the tumor subsided >1 cm distal to the bronchial opening of the right upper lobe. No cancer was observed during the surgery. On postoperative pathological reports, the size of the lung tissue was 12 cm × 10 cm × 5.5 cm, and the size of the tumor bed was 3 cm × 2 cm × 2 cm. Under a microscope, necrosis, histiocyte reaction, cholesterol crystal, and inflammatory cell reaction were observed. No residual tumor cells were found, in line with the post-treatment reaction (Figure 4c and d). Immunohistochemistry showed CK (−), CD68 (histiocyte +), and p63 (−). No cancer cells were observed in the bronchial stump.

**Informed consent:** Informed consent has been obtained from all individuals included in this study.**Ethical approval:** The research related to human use has been complied with all the relevant national regulations, institutional policies and in accordance with the tenets of the Helsinki Declaration, and has been approved by the author's institutional review board or equivalent committee.

## 3 Discussion

This case report describes a case with resectable NSCLC treated with neoadjuvant chemotherapy (paclitaxel + carboplatin) + immunotherapy (durvalumab) followed by surgery.

Neoadjuvant chemotherapy combined with durvalumab achieved a satisfactory CPR demonstrating preliminary promise for this treatment strategy. These findings are consistent with the preliminary results of previous studies [16,18,19,20]. Furthermore, in a single-arm, phase I trial with neoadjuvant nivolumab before pulmonary resection in patients with resectable NSCLC (*N* = 22), the major pathological response (MPR) in the resected tumor was 45%, and recurrence-free survival was 73% after 18 months of surgery [18]. Hence, neoadjuvant nivolumab monotherapy was feasible and safe in patients with resectable early NSCLC and provided clinical evidence for further research.

Further, a few more studies were carried out to explore the effect of immunotherapy combined with chemotherapy at neoadjuvant settings [16,19,20]. An open-label, multicenter, single-arm phase 2 trial (NADIM trial) from Spain evaluated the efficacy of neoadjuvant chemotherapy (paclitaxel + carboplatin) plus nivolumab immunotherapy in patients with resectable stage IIIA NSCLC (*N* = 46) before surgery [16,19]. Around 83% of the patients had MPR, 71% of the patients achieved CPR, and tumor downstaging was observed in 90% of the patients after neoadjuvant treatment with immunotherapy plus chemotherapy [19]. The recent results of the NADIM trial showed PFS in 77.1% of the patients at 24 months of follow-up without any adverse event associated with surgery delay or death [16]. Yet another open-label, multicenter, single-arm, phase 2 trial from the USA evaluated the efficacy of immunotherapy + chemotherapy (atezolizumab, with carboplatin and nab-paclitaxel) as a neoadjuvant treatment before surgical resection in patients with resectable stage IB–IIIA NSCLC (*N* = 30) [20]. A high proportion of the patients (57%) achieved MPR during 12.9 months of median follow-up period with manageable adverse events. However, no studies are evaluating durvalumab in the neoadjuvant setting for NSLC to our knowledge. Hence, our case study added evidence for the chances of a cure for a potentially lethal locally advanced NSCLC by choosing neoadjuvant durvalumab + chemotherapy for the treatment. However, there is no standard preoperative neoadjuvant treatment course, and the treatment course of two to three cycles has been selected based on the previous studies.

In phase 3 PACIFIC trial, patients with stage III unresectable NSCLC treated with durvalumab in adjuvant settings showed significantly longer progression-free survival and overall survival than patients treated with placebo [7,15]. PACIFIC trial changed the management of stage III NSCLC establishing durvalumab as a standard of care in these patients [21]. However, there is no sufficient evidence on the efficacy of durvalumab + chemotherapy as neoadjuvant treatment in patients with resectable stage III NSCLC. The ongoing trials investigating the efficacy of neoadjuvant durvalumab in stage III NSCLC are yet to publish the final results (NCT02572843, NCT03800134). Hence, our case study provides early clinical evidence for achieving CPR in a patient with resectable stage III NSCLC on treating with neoadjuvant immunotherapy (durvalumab) combined with chemotherapy before surgery.

After considerable regression of the tumor in the patient, we opted for thoracoscopic upper lobectomy and lymph node dissection as the pulmonary edema was mild and the separation of tissue space was not difficult. In the neoadjuvant chemotherapy era, sleeve resection or lobectomy plus bronchoplasty was a reasonable surgical plan in the case of tumor growth and infiltration to the bronchial orifice before neoadjuvant therapy. At present, there is no definite conclusion about the clinical scope of lobectomy. However, in this case, we performed routine lobectomy and final pathological examination of the frozen bronchial resection edge. As the resected bronchial margin was negative for the tumor, we did not perform sleeve resection of the upper lung.

Further, there is no clear guidance in clinical practice for postoperative adjuvant treatment in patients with resectable stage III NSCLC. However, based on the study NADIM trial, we can consider immunoadjuvant therapy for 1 year after surgery or conventional adjuvant chemotherapy for 4 courses [16].

Further, based on the results of preliminary studies and our case study, it is still unclear whether neoadjuvant immunotherapy combined with chemotherapy can improve long-term patient prognosis. Hence, long follow-up results of these studies are warranted to confirm the efficacy and safety of neoadjuvant immunotherapy combined with chemotherapy in patients with resectable stage III NSCLC.

## 4 Conclusion

In this study, patients with resectable stage III NSCLC treated with neoadjuvant chemoimmunotherapy achieved a complete pathological response, thus providing clinical evidence for future research. However, advanced phase 3 studies with long-term follow-up are imperative to provide further evidence for the chances of cure for a potentially lethal locally advanced resectable NSCLC.
